# NLRP3 and Infections: β-Amyloid in Inflammasome beyond Neurodegeneration

**DOI:** 10.3390/ijms22136984

**Published:** 2021-06-29

**Authors:** Giulia Sita, Agnese Graziosi, Patrizia Hrelia, Fabiana Morroni

**Affiliations:** Department of Pharmacy and BioTechnology—FaBiT, University of Bologna—Alma Mater Studiorum, via Irnerio 48, 40126 Bologna, Italy; giulia.sita2@unibo.it (G.S.); agnese.graziosi2@unibo.it (A.G.); fabiana.morroni@unibo.it (F.M.)

**Keywords:** NLRP3, neuroinflammation, infections, neurodegeneration, Alzheimer’s disease, COVID-19

## Abstract

Amyloid beta (Aβ)-induced abnormal neuroinflammation is recognized as a major pathological feature of Alzheimer’s disease (AD), which results in memory impairment. Research exploring low-grade systemic inflammation and its impact on the development and progression of neurodegenerative disease has increased. A particular research focus has been whether systemic inflammation arises only as a secondary effect of disease, or it is also a cause of pathology. The inflammasomes, and more specifically the NLRP3 inflammasome, are crucial components of the innate immune system and are usually activated in response to infection or tissue damage. Although inflammasome activation plays critical roles against various pathogens in host defense, overactivation of inflammasome contributes to the pathogenesis of inflammatory diseases, including acute central nervous system (CNS) injuries and chronic neurodegenerative diseases, such as AD. This review summarizes the current literature on the role of the NLRP3 inflammasome in the pathogenesis of AD, and its involvement in infections, particularly SARS-CoV-2. NLRP3 might represent the crossroad between the hypothesized neurodegeneration and the primary COVID-19 infection.

## 1. Alzheimer’s Disease and Inflammation

Alzheimer’s disease (AD) is the most common neurodegenerative disease in older people representing the most frequent form of dementia worldwide [1]. Although by 2050, the number of diagnoses is expected to reach 131.5 million, to date, almost all clinical trials have failed—underling the need for further research of novel therapeutic approaches [2].

AD is mainly characterized by two pathological changes, the deposition of extracellular senile plaques of β-amyloid (Aβ) protein, and the formation of intracellular neurofibrillary tangles (NFTs), due to the hyperphosphorylation of tau protein [1]. In physiological conditions, Aβ peptides are removed from the brain tissue both by degradation and by removal into cerebrospinal fluid and blood vessels [3].

The abnormal accumulation of Aβ in the brain, especially the oligomeric form, is an early feature of AD and is usually associated with neuronal loss, inflammatory responses, and oxidative stress [4]. Recently, increasing evidence suggests a central role of the immune system in the progression or even the origin of AD [5]. Many studies highlighted the role of neuroinflammation in the progression of AD, in which the release of inflammatory mediators can influence neuronal cells and their function [6]. Indeed, the inflammatory response has a crucial role in different neurodegenerative diseases, and it is primarily driven by the microglia, which release cytokines causing chronic neuroinflammation [6,7]. Microglia originate from primitive macrophages and are the resident myeloid cells of the central nervous system (CNS). These cells not only check for pathogens and cell debris in the parenchyma of the CNS, but also support homeostasis and brain plasticity, being involved in the formation of neuronal connections during development [8,9,10]. In physiological conditions, microglia play a critical role in several developmental events, as the formation of neural circuits, synaptic pruning, and remodeling, neurogenesis, clearing cellular debris, proteins aggregate, and pathogens [3,11,12,13]. Microglia are the non-neuronal CNS cells most closely related to changes observed in AD. Microglia remove by phagocytosis Aβ oligomers and protofibrils [14]. Aβ oligomers propagate into the brain parenchyma, arousing a stronger microglial response and memory impairment than fibrils [15]. Microglia immune response against Aβ oligomers in the hippocampus may be implicated in the pathogenesis of late-onset AD [16]. However, impairment in this capacity may lead to an adverse increase of Aβ species in the CNS, which could then aggregate further into Aβ plaques. Moreover, Aβ peptide can bind to microglia’s receptors driving the production of proinflammatory cytokines and chemokines [17,18]. Different studies have shown in AD mouse models that the increase of CD68, a marker for microglial activation, is associated with Aβ plaques [19,20]. Furthermore, the fibrillar form of Aβ can induce inflammasome activation in microglia, but less is known about the capacity of small Aβ oligomers and protofibrils, that seem to be more neurotoxic, to activate the inflammasome in microglia [21,22]. The extended exposure of microglia to Aβ can impair their function, decreasing phagocytosis and reducing the capacity to extend processes towards the lesioned tissue with the result that Aβ is inefficiently cleared from the neuronal tissue [23,24]. Different proinflammatory cytokines, like the tumor necrosis factor–α (TNF–α) or interleukin (IL)–1β, –6, –12 and –23, maintain a state of microglial activation and they could trigger each other, leading to a positive feedback loop which accelerates AD pathology [25,26,27]. In particular, IL-1β induces IL-6, which is dramatically increased in AD patients [28]. This last cytokine may have a crucial role in AD, indeed not only a mutation in the gene encoding for IL-6 may result in a late––onset disease, but it can also play a role in the synthesis and expression of amyloid precursor protein (APP) [29]. Moreover, higher levels of IL-1β may affect tau hyperphosphorylation, and thus, aggravate AD pathology, impairing long term potentiation (LTP) and memory formation [30]. In physiological conditions, microglia have a critical role in maintaining a healthy brain. Some structural variants of genes expressed on microglia and encoding for immune receptors, such as TREM2, CD33, and CR1, have been associated with a higher risk of AD [31,32,33]. Moreover, altered gene expression in the regulation of the immune system in AD and its contribution to the pathology, support a pathogenic role of CNS–resident myeloid cells, like microglia, in the evolution of the disease [34,35]. Microglia dysfunction may occur not only by mutations, but also consequently to a long–lasting Aβ exposure [36].

Regardless of the cause, altered microglia lose their beneficial and physiological functions to develop a detrimental, senescent–like phenotype. In addition, when microglia become overactivated or reactive, they can induce detrimental neurotoxic effects releasing numerous cytotoxic elements [37].

Besides the activation of microglia by Aβ, as well as NFTs, AD evolution may be influenced by the activation of pattern recognition receptors (PRRs), which can detect different pathogens or pathogen/danger–associated molecular patterns (PAMPs or DAMPs) [38,39]. PRRs can be divided into two major classes depending on their subcellular localization. The membrane proteins, named Toll–like receptors (TLRs) and C–type lectin receptors (CLRs), which can recognize extracellular PAMPs and DAMPs. The second class of PRRs is intracellularly located and includes RIG–I–like receptor (RLR), HIN–200 family member AIM2–like receptor (ALR), and NOD–like receptor (NLR) proteins [40]. NLRs and ALRs can form complexes, named inflammasomes, responsible for a strong inflammatory response to cellular infection and stress [41]. Under physiological conditions, inflammasome is inactive in the cytoplasm, and it cannot oligomerize without an activating signal [42]. On the contrary, when inflammasome receptors recognize PAMPs or DAMPs by damaged cells and pathogens, a multiprotein complex is formed [43]. Once activated, inflammasomes drive the secretion of the proinflammatory cytokines IL-1β and IL-18, IL-1α and HMGB1 alarmins, and ultimately, pyroptotic cell death [44,45]. Pyroptosis is an inflammatory programmed cell death pathway that takes place in T–lymphocytes and results in cellular events, including swelling of the cytoplasm, plasma membrane rupture, and consolidation of the nucleus with the release of cytoplasmic contents into the extracellular space [46]. This type of cell death is activated through gasdermin D (GSDMD) cleavage by caspase-1, -4, -5, which stimulates IL-1β and IL-18 secretion [47]. The secretion of proinflammatory cytokines IL-1β and IL-18 is mainly induced by microglia and astrocytes, and drives the inflammatory responses contributing to the neuronal damage and the phagocytic capacity of microglial cells [45,48].

**Figure 1:**
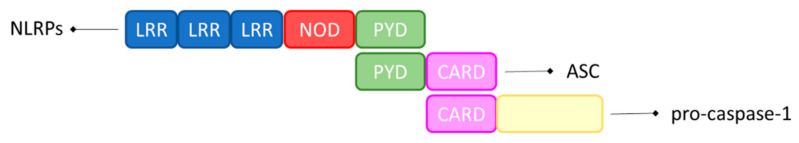
Schematic representation of the inflammasome’s components. Inflammasome contains a C–terminal LRR domain, an N–terminal CARD or PYD domain, and a central NOD domain. ASC consists of an N–terminal PYD and a C–terminal CARD. Once activated, inflammasome acts as a sensor molecule and connects to ASC via the PYD–PYD interaction. Finally, ASC recruits pro-caspase-1 via CARD–CARD interaction, which promotes the self–cleavage and the activation of pro-caspase-1.

Inflammasomes are defined as ‘canonical’ when their assembly requires caspase-1, and as ‘non-canonical’ when their assembly depends on human caspase-4 or caspase-5. As shown in Figure 1, proteins in the NLR family are constituted of a central NOD domain, C–terminal leucine–rich repeats (LRRs), and N–terminal caspase recruitment domains (CARD) or pyrin domains (PYD). These sensors initiate the assembly of canonical inflammasomes by recruiting caspase-1, with or without the ASC adaptor in an ATP–dependent manner [39].

Microglia express several PRRs, which detect modifications in the CNS environment through recognizing DAMPs and PAMPs and send converging signals to promote a spectrum of microglial responses from surveillance to activation [40]. Once activated, microglia assume different morphologies, and produce several cytotoxic molecules, including proinflammatory cytokines, inflammatory mediators like nitric oxide (NO), and reactive oxygen species (ROS) [49]. Among inflammasomes, NLRP3 is the most studied and characterized, and its activation in neurodegenerative diseases mainly focuses on astrocytes and microglia [50]. This review presents an overview of the findings in current research focusing on NLRP3 activation and the possible implication in AD.

## 2. NLRP3 and Aβ

NLRP3 is ubiquitously expressed in CNS, and it has been found to be highly expressed in the brain of AD patients [43]. To date, no data is available about the possible pharmacological approach in AD based on the inhibition of NLRP3. Misfolded protein aggregates, like Aβ depositions, can promote NLRP3 activation by increasing the expression of the major histocompatibility complex II (MHC–II) on the cell surface [21].

After its activation, the NLRP3 inflammasome increases the release of active caspase-1, and the subsequent secretion of IL-1β and IL-18, which may result in chronic inflammatory responses, neuronal death, and pyroptosis in the CNS [51]. On the other hand, the inhibition of IL-1β signaling may contribute to disease–modifying effects, as shown by the expression of IL-1β in Aβ–plaque, associated with microglial cells [52,53]. Evidence suggests that the levels of active caspase-1 and IL-1β increase in the microglia of AD animal models and patients, and it can be associated with the onset and progression of the pathology [21,27,54]. Moreover, patients with amyloidosis showing cognitive impairments present higher levels of proinflammatory cytokines and lower levels of IL-10 in the serum, as compared to patients without brain amyloidosis [55]. In this view, the activation of microglia in the hippocampus may influence the cytokine profiles in the serum, and this activation may result in a decrease of IL-10 levels [56]. Indeed, emerging evidence indicates that IL-10 may act in a negative feedback loop to regulate the NLRP3 inflammasome during chronic stimulations [57].

An interesting study by Lučiūnaité et al. shows that soluble, low molecular weight Aβ oligomers and protofibrils, with a maximum size of 5 nm, can activate the NLRP3 inflammasome in microglia [58]. The study shows that small Aβ fragments activate murine microglia without altering their viability, suggesting that these species can induce an innate immune response prior to their deposition in amyloid plaques. Moreover, the authors investigate whether soluble Aβ species were able to activate the NLRP3 inflammasome. Indeed, as fibrillary Aβ can act as DAMP and activate the NLRP3 inflammasome, also small soluble Aβ oligomers and protofibrils may activate all components of the NLRP3 inflammasome too, inducing an early neuroinflammatory response [58,59]. Additionally, the activation of the NLRP3 inflammasome boosts Aβ aggregation by reducing phagocytosis [54].

In AD, the presence of Aβ plaques recruits microglia to phagocyte the aggregated forms, especially oligomers and fibrils. This condition induces the activation of the NLRP3 inflammasome, with a subsequent release of proinflammatory cytokines, as IL-1β, and potentially neurotoxic factors. In turn, cytokines and factors released to enhance the neurotoxic effects of Aβ and worsen the pathological processes of AD [50]. It has been described that the NLRP3 inflammasome might be essential for the immune responses in AD. Indeed, Halle et al. showed that Aβ increased the activation of the NLRP3 inflammasome in microglial cells [21]. This hypothesis is also confirmed by Heneka et al., who showed in APP/PS1 mice, that Aβ can activate NLRP3 inflammasome in microglia, inducing an inflammatory M1 phenotype, characterized by an elevated expression of proinflammatory factors, resulting in increased hippocampal and cortical Aβ deposition, neuronal loss, and cognitive impairment. Interestingly, NLRP3 activation in APP/PS1 mice occurs only in microglia associated with the presence of Aβ plaques depositions; underlying that microglia–specific NLRP3 activation contributes to AD pathogenesis. However, in this transgenic model, mice with deletions for NLRP3 or caspase-1 show a reduced impairment in spatial memory abilities and a lower inflammatory response. Moreover, the deletion induces microglia anti-inflammatory M2 phenotype, with decreased caspase-1 and IL-1β secretion, reduced amyloid depositions, and improved cognitive functions [54,60].

Another study conducted by Venegas et al. on APP/PS1 mice showed that the intrahippocampal injection of ASC fragments promotes Aβ plaque formation and accumulation, but it failed to induce Aβ pathology in ASC–deficient mice [49]. Moreover, ASC or NLRP3 deficiencies have been associated with a decreased tau pathology and protected tau transgenic mice against cognitive impairment [61]. Another study has shown that the suppression of IL-1β in the triple transgenic (3 × Tg) mouse model of AD restores cognitive abilities, reduces tau pathology, and reestablishes the function of the neuronal beta–catenin pathway [52]. In this view, the specific abnormal activation of NLRP3 in microglia induces chronic neuroinflammation in AD, leading to microglial Aβ phagocytic dysfunction and neuronal damage. However, this process might be altered by a damage to the inflammasome. In summary, clarifying the links between innate immune activation and microglia–dependent NLRP3 inflammasome activation may explain the functional role of NLRP3 in AD. Its regulation may reduce neuroinflammation in AD, and therefore, be a novel therapeutic strategy for this disease.

A recent study demonstrates that NLRP3 inflammasome plays a critical role in a mouse model of sporadic AD. Results showed that the intracerebroventricular (icv) injection of streptozotocin (STZ) activated the NLRP3 inflammasome, reduced Aβ clearance, and induced neuronal loss and cognitive impairment. Moreover, the inflammatory response enhanced the activation of NLRP3, amplifying the microglial reaction, and worsening the pathological damage. Interestingly, the inhibition or depletion of microglial NLRP3 reversed these effects [62].

In a recent study, Fekete et al. investigated the microglial response and mechanisms induced by Aβ oligomers in an early rat model of AD [56]. These authors showed not only that microglia in the hippocampus is activated four weeks after oligomers infusion, but also that chemokines, complement, and proinflammatory cytokines were upregulated, indicating the activation of the nuclear factor kappa–light–chain–enhancer of activated B cells (NF–ĸB). This transcription factor is a key regulator of the inflammatory response, and its activation stimulates the β–secretase (BACE1) cleavage of APP and consequently Aβ production [63]. Moreover, the inhibition of NLRP3 inflammasome by icv infusion of a known inhibitor, MCC950, reduced microglia reactivity, NF–ĸB activation, and spatial memory impairment [56]. Interestingly, this study showed that the presence of Aβ oligomers, without amyloid plaques formation, is necessary to induce an inflammatory response and memory deficits in an NLRP3–dependent manner. Therefore, the inhibition of NLRP3–inflammasome could represent a new therapeutic approach.

As known, NLRP3 inflammasome can be activated by Aβ, leading to IL-1β overproduction, neuroinflammation, and cognitive impairment [64]. In this view, Lonneman et al. investigated the potential inhibition of the NLRP3 inflammasome and the subsequent reduction of IL-1β production with dapansutrile in an APP/PS1 mouse model of AD [65]. Dapansutrile is a small molecule that targets the NLRP3 inflammasome preventing the activation of caspase-1 and the release of IL-1β [66]. Results obtained suggested the possible benefits of delaying NLRP3 signaling in AD, indeed after the treatment, APP/PS1 mice improved cognitive functions with a reduction in proinflammatory cytokines in the brain. Furthermore, the authors observed an increased phagocytic capacity of microglia in the cortex of APP/PS1 mice treated with the compound in the study as compared to control animals [65].

Ising et al. demonstrated that the effect of the NLRP3 inflammasome in AD may be considered as a promising target for treating the pathology [61]. Indeed, inhibiting the NLRP3 inflammasome decreased tau phosphorylation and aggregation, while fibrillar Aβ facilitated tau pathology by activating NLRP3 [67]. Results obtained in transgenic Tau22/Asc^−/−^ and Tau22/Nlrp3^−/−^ mice showed a considerable reduction of tau phosphorylation with reduced cognitive decline, which indicates that NLRP3 is an important mediator of Aβ–induced tau pathology [61].

All these findings highlighted a fundamental role of the NLRP3 inflammasome in the progression of AD and suggested that the pharmacological inhibition of the NLRP3 may represent a turning point in treating neurodegenerative diseases.

## 3. NLRP3 and Infections

A multitude of viruses can cause severe diseases, such as hepatitis C virus (HCV), human immunodeficiency virus–1 (HIV–1), and influenza A virus (IAV). For this reason, the host has evolved highly conserved sensors, named PRRs, to remove invading viruses activating antiviral immune response [68]. Moreover, the role of the NLRP3 inflammasome is essential for the antiviral immune responses. Indeed, several viruses induce early activation of NLRP3, which reduces viral replication and decreases mortality in mouse models [69]. In physiological conditions, NLRP3 levels are low to prevent aberrant inflammasome activation. In the case of viral infection, NF–κB signaling is activated through PRRs–dependent pathways, which induce IFN–β or TNF–α activation that, in turn, activate NF–κB to initiate the NLRP3 inflammasome response [70]. To the best of our knowledge, a specific ligand able to bind directly to NLRP3 is not known. The activation of the inflammasome is usually associated with PAMPs and DAMPs. Interestingly, also small viral components could activate the NLRP3 inflammasome inducing IL-1β secretion in macrophages [71]. ROS formation and cellular homeostasis are fundamental for the NLRP3 inflammasome activation, as potassium (K^+^) or calcium (Ca^2+^) efflux or influx are established activators that lead to mitochondria damage and ROS formation, potentiating the NLRP3 inflammasome activation [72,73,74].

We have already explained as Aβ formation may induce the NLRP3 inflammasome activation. Interestingly, the open reading frame 8b (ORF8b) of the severe acute respiratory syndrome coronavirus–2 (SARS–CoV–2) forms intracellular aggregates that represent a danger signal able to induce endoplasmic reticulum stress and lysosomal damage, resulting in the NLRP3 inflammasome activation [75]. Several studies showed that the NLRP3 inflammasome and IL-1β are implicated in the inflammatory response during lung injury and acute respiratory distress syndrome (ARDS) [76,77]. Indeed, middle east respiratory syndrome–related coronavirus (MERS CoV), SARS–CoV, and influenza patients with ARDS not only show higher levels of IL-1β in bronchoalveolar fluid and plasma as compared to healthy controls, but this condition is also associated with worse clinical outcomes [78,79,80]. Indeed, the aberrant activation of NLRP3 and downstream mediators often lead to pathological tissue injury during infection [81]. For example, several studies have highlighted its important role in relation to the pathogenesis of ARDS, which is driven by the same proinflammatory cytokines released by the inflammasome [82]. Interestingly, animals lacking in inflammasome’s components showed reduced lung injury and increased survival rate following influenza infection [83]. A recent study by Blanco–Melo et al. demonstrated that SARS–CoV–2 infection induced the expression of many cytokines and chemokines, including TNF–α, IL-6, and IL-1β, contributing to the tissue damage [84]. Even more interestingly, this pathological immune response is characterized by a hyperinflammatory microenvironment limited to the site of tissue injury. With the development of the inflammatory cascade, IL-1β and TNF–α induce the secretion of further NLRP3 cytokines, such as IL-6, which, owing to the loss of vascular integrity, can be detected in the peripheral blood and may activate the NLRP3 inflammasome in other immunological pathways [85,86].

In conclusion, NLRP3 activation and associated inflammation are a double–edged sword in the antiviral host defense. The modulation of NLRP3 inflammasome activity may be a promising approach to counteract viral diseases and the subsequent inflammatory reactions.

## 4. Aβ and Infections

Antimicrobial peptides (AMPs) are cationic, amphiphilic, and α–helical molecules, which represent the first-line defense of the innate immune system in different species, including human [87]. AMPs are widely expressed in the brain, and although their function is normally protective, their dysregulation can lead to cell toxicity, chronic inflammation, and degenerative pathologies [88]. Viruses can affect upstream molecular pathways to indirectly drive Aβ depositions or induce Aβ formation by interacting directly with the viral surface or specific viral proteins. Indeed, AMPs share many characteristics with Aβ protein, as oligomerization and fibrillization, that mediate key protective roles in innate immunity [89]. Soscia et al. demonstrated in 2010 that Aβ peptides show antimicrobial activity in in vitro models towards different common and clinically relevant microbial pathogens [90]. In particular, the authors showed not only that Aβ_1–42_ potency was greater compared to Aβ_1–40_, but also confirmed that the antimicrobial activity is peptide-specific and no effects were observed after the treatment with reverse (rAβ_1–42_) or scrambled (scAβ_1–42_) negative control peptides [90]. According to these findings, viral infections may contribute to initiate or accelerate Aβ accumulation in the brain, leading to AD. Moreover, the consequent inflammatory response activated in the CNS could evolve into an aberrant immune response induced by the persistent accumulation of cerebral Aβ, which clearly contributes to AD pathogenesis. Kumar et al. showed that Aβ expression could inhibit infection in a transgenic mouse model of AD (5XFAD), in the nematode *Caenorhabditis elegans* (*C. elegans*) model and in cultured mammalian cell models. Most surprisingly, oligomerization and fibrillization seem to mediate Aβ protective activity, and dramatically accelerates Aβ deposition in 5XFAD mice and in transgenic *C. elegans* [88].

Several studies have indagated the interaction between amyloids and Herpes simplex-1 (HSV-1) in AD patients [91]. HSV-1 can enter the CNS through the trigeminal ganglion, where it can both remain in a latent state or cause acute encephalitis. Different findings highlighted that the periodic reactivation of HSV-1 during the lifespan can trigger the molecular mechanisms leading to AD. In 2018, Tzeng et al. conducted a study involving 33,000 patients monitored for 16 years, which showed that HSV-1 infections can be correlated to an increasing AD risk of 2.5-fold [92]. Similarly, Herpes simplex-2 (HSV-2) infection was shown to induce accumulation of hyperphosphorylated tau and Aβ depositions [93]. HSV viruses are not isolated cases of correlation between infections and amyloids, indeed varicella–zoster virus (VZV), cytomegalovirus (CMV), human beta–herpes virus 6A and 6B (HHV-6A and -6B) infections could increase the risk of AD [94,95,96]. Finally, White et al. showed in vitro the antiviral activity of Aβ peptide against H3N2, H1N1, and IAV virus by inducing virion aggregation, and reducing the infection rate [97].

Currently, the major concerns are related to the possible future neurodegenerative consequences following the new coronavirus disease 19 (COVID-19) infection. Systemic inflammation, through activation of the NLRP3 inflammasome, impairs immune homeostasis in the brain, promoting the production of proinflammatory cytokines as IL-1β [98]. In these conditions, Aβ peptides (or other peptides) aggregate in fibril form, block viral particles, promoting the viral inflammatory response and contributing to neurodegeneration, as in AD and Parkinson’s disease (PD) [61,99]. The NLRP3 inflammasome might represent the crossroad between SARS–CoV–2 infection and neurodegenerative outcomes depending on numerous factors, like the extension of the ground inflammatory response or the leukocytes invasion/migration to the brain [100].

## 5. CoVs Infections and Neurodegeneration

Some evidence indicated that SARS–CoV–2, as MERS–CoV and SARS–CoV, can remain latent with potential long term and considerable effects on neurological function—for instance, viral infections may be related to extrapyramidal symptoms and pathological modifications in substantia nigra and other subcortical nuclei [101,102,103]. Indeed, several respiratory virus infections have been shown to propagate in cerebral regions associated with learning and cognitive abilities [104]. This condition could silently initiate or accelerate a neurodegenerative process lasting decades before symptoms being manifest, reminding AD. At present, it is not possible to establish any correlation between COVID-19 and AD development, but animal studies have confirmed tau pathology related to inflammation induced by a viral infection and associated with cognitive impairments [105]. For this reason, it is interesting to study if COVID-19 infection may represent a risk to develop neurodegenerative diseases that should be experimentally proved in the next future [106].

It is already demonstrated that the mechanism by which SARS–CoV–2 infiltrates the cells is mediated by the binding with Angiotensin–Converting Enzyme 2 (ACE2), a transmembrane protease mainly present in the respiratory tract, vascular endothelium, kidney, small intestine, and brain [107]. The preference of the virus for the tissues expressing ACE2 could mean that all the conditions involving the upregulation of ACE2 expression may increase the risk of virus invasion and potentially contribute to molecular processes leading to neurodegeneration. For instance, NO, a key neurotransmitter in cognitive functions, could be lowered in neurons following the binding of SARS–CoV–2 with the vasoconstrictor type 1 angiotensin II receptor (AT1R) through overexpressed ACE2 [108]. Therefore, COVID-19 patients would be more vulnerable to behavioral and cognitive decline [109]. To date, the mechanism by which SARS–CoV–2 may invade the CNS is still unclear, although some similarities can be found with other viruses and CoVs.

Interaction of the SARS–CoV–2 with ACE2 receptor induces the neuroinflammatory cascade, and the blood brain barrier (BBB) becomes compromised. In the human brain, ACE2 expression may be considered relatively high and selective for specific brain areas, such as substantia nigra and brain ventricles [110]. This infection might slow down respiratory regulation, which, combined with decreased lung activity, may result in a hypoxic condition [111]. Hypoxia and neuroinflammation could compromise the cortical and hippocampal structures, causing neurological disorders [112]. Accordingly with this hypothesis, RNA virus and inflammatory markers have been detected in cerebrospinal fluid (CSF) of COVID-19 patients [113]. On the other hand, peripheral inflammation may direct neurologic symptoms. Indeed, high levels of cytokines can pass directly or compromise BBB integrity allowing the diffusion of the virus–infected monocyte leading to neuroinflammation [114]. The dysregulation of immune response is recognized as a key mechanism involved in neurodegeneration, and increased levels of IL-1β and IL-6 are frequent in patients, indeed neuroinflammation can accelerate neurodegenerative processes in the pre-clinical stages [114,115].

Increasing evidence shows that CoVs may firstly invade peripheral nerve terminals to then get access to the CNS through synapses or via the olfactory nerves [116]. Indeed, the expression of ACE2 and transmembrane serine protease 2 (TRPMSS2) receptors, the presumed receptors for the virus, abundant in nasal epithelial cells, are increased following the virus infection, which could be disseminated in the CNS via axonal transport [117]. Moreover, olfactive dysfunction is also an early event in neurodegenerative diseases like AD [118]. Moreover, the enteric nervous system and vagus nerve are considered a potential path to entry in CNS, indeed in patients with PD, α–synuclein pathology may follow the same progression from the enteric nervous system to the brain [119,120].

Neuroinflammation represents a key point in neurodegeneration and in AD evolution, during which inflammatory cytokines levels have been reported to be related to disease progression [121]. Plasma levels of IL-6 were significantly increased in 47 AD patients compared to healthy individuals at the same age [122]. In line with this, a recent study on mice evaluating memory and learning capacities reveals that the animals’ knockout for IL-6 showed a better cognitive performance [123]. In this view, IL-6 may represent a pleiotropic biomarker for AD and COVID-19, in CNS and respiratory system, respectively.

Recently, in the UK Biobank Community Cohort, the apolipoprotein E ε4 (APOEε4) homozygous genotype, typically associated with an increased risk of AD, was also found to be associated with an increased risk of severe COVID-19 infection [124]. APOE ε4 has also been related to BBB leakage and cerebral amyloid angiopathy [125]. The biological mechanism behind this association needs to be further investigated, indeed APOEε4 has a strong influence on neuronal inflammation, potentially spreading pathological proteins through the brain and leading to AD exacerbation [106].

## 6. Conclusions

Neuroinflammation is a crucial factor that contributes to AD pathology. Several studies investigated the efficacy of anti-inflammatory therapeutic approaches to change the course of the disease [6]. To date, the role of Aβ peptide is still not clear; however, a new proposal for its activity investigates whether it is involved as AMPs. Thus, physiological protective activity of Aβ may conduct to AD pathology once dysregulated. In this view, Aβ may protect against infections, but can also lead to propagating pathological amyloidosis.

Moreover, inflammation, due to infections, is driven by the NLRP3 activation through PAMPs and DAMPs. Besides this, the NLRP3 inflammasome activation plays a fundamental role also in the immune response and in neurodegenerative disease, as AD. Indeed, Aβ can act as DAMP and activate the NLRP3 inflammasome, inducing an early neuroinflammatory response.

At present, a great concern is about the potential neurodegenerative consequences of SARS–CoV–2 infection. Direct or indirect invasion into CNS induces the production of cytokines, activates microglia, and can contribute to the propagation of neurodegenerative processes. On the other hand, viral replication can also promote unfolded protein response, and trigger misfolded protein accumulation. Many features are shared between COVID-19 and neurodegenerative diseases, which may underlie the higher risk for patients to develop neurodegenerative diseases, as AD, in the future. The next few years will be crucial for identifying potential early indicators of neurodegeneration in patients who have survived COVID-19 and to finally elucidate the physiological and pathological role of Aβ and the NLRP3 inflammasome.
